# Prescriptive Target Matching Across Hearing Aid Technology Generations: A Real-Ear Insertion Gain Analysis

**DOI:** 10.3390/audiolres16050145

**Published:** 2026-09-25

**Authors:** Megan Kruger, Vinaya Manchaiah, Steve Taddei, Abram Bailey, De Wet Swanepoel

**Affiliations:** 1 Department of Speech-Language Pathology and Audiology, University of Pretoria, Pretoria 0028, South Africa; megan.knoetze@up.ac.za (M.K.); vinaya.manchaiah@cuanschutz.edu (V.M.); 2 Virtual Hearing Lab, Collaborative Initiative between University of Colorado School of Medicine (USA) and the University of Pretoria (South Africa), Aurora, CO 80045, USA; 3 Department of Otolaryngology-Head and Neck Surgery, University of Colorado School of Medicine, Aurora, CO 80045, USA; 4 UCHealth Hearing and Balance, University of Colorado Hospital, Aurora, CO 80045, USA; 5 Department of Speech and Hearing, School of Allied Health Sciences, Manipal Academy of Higher Education, Manipal 576104, India; 6 Hear Advisor LLC, Rockford, IL 61102, USA; steve@hearadvisor.com (S.T.); abram@hearingtracker.com (A.B.); 7 Hearing Tracker Inc., Austin, TX 40702, USA

**Keywords:** hearing aids, prescriptive target matching, real-ear insertion gain, real-ear measurements, technology generation

## Abstract

**Background:** Advances in hearing aid technology may improve prescriptive target matching, but whether newer devices better match targets remains unclear. This study compared prescriptive target matching across hearing aid technology generations. **Methods:** This secondary analysis included 29 flagship prescription hearing aids grouped into older (2021–2022; n = 8), mid (2023; n = 11), and newest (2024–2025; n = 10) technology generations. Root Mean Square Error (RMSE) between measured Real Ear Insertion Gain (REIG) and NAL-NL2 targets was calculated across six frequencies (500–6300 Hz) for soft (55 dB SPL), conversational (65 dB SPL), and loud (75 dB SPL) speech levels under Initial Fit and Tuned Fit conditions. Kruskal-Wallis tests and linear mixed-effects models accounting for manufacturer clustering were used to evaluate prescriptive target matching across technology generations. **Results:** No significant technology-generation differences were observed at Initial Fit. Following tuning, group comparisons showed differences at conversational (*p* = 0.006; ε^2^ = 0.321) and loud (*p* = 0.004; ε^2^ = 0.347) levels, with a directionally consistent but non-significant difference at soft speech (*p* = 0.055; ε^2^ = 0.146). After accounting for manufacturer clustering, release year remained a significant predictor of closer target matching only at conversational speech (β = −0.15 dB/year, 95% CI [−0.29, −0.01], *p* = 0.048). Sensitivity analyses supported the robustness of this finding. **Conclusions:** Newer hearing aids showed closer prescriptive target matching following tuning at conversational speech, although effects at soft and loud speech did not survive adjustment. Technological advances may improve prescriptive target matching at conversational input levels during fitting.

## 1. Introduction

Prescription hearing aids are typically fitted according to prescriptive formulae, such as the National Acoustic Laboratories–Non-Linear 2 (NAL-NL2) procedure, which specify frequency-specific gain targets intended to maximize speech audibility while limiting loudness discomfort [1]. Real-ear measurement (REM) verification is recommended in clinical practice to confirm that a hearing aid’s output matches prescribed targets following hearing aid fitting [2]. A systematic review and meta-analysis found that REM-verified fittings were associated with moderate improvements in speech intelligibility, sound quality, and patient preference relative to manufacturers’ unverified default settings [2]. Clinic-based data have similarly linked REM verification to closer prescriptive target matching and greater patient loyalty [3]. Moreover, REMs have generally been included in recommended clinical protocols for hearing aid fitting and management [4,5]. Consequently, achieving close prescriptive target matching remains an important component of best-practice hearing aid fitting.

Despite these benefits, REM verification is not universally performed, and hearing aids are often initially fitted using manufacturer-recommended fitting approaches without probe-microphone verification [6,7]. Whether these approaches provide an adequate match to prescriptive targets depends, in part, on how accurately manufacturers’ fitting software approximates these targets, which has historically been a key rationale for routine REM verification [2]. More recent evidence, however, suggests that contemporary fitting software has narrowed this gap. For example, a registered clinical trial found that default manufacturer fittings were sufficiently close to real-ear-verified targets that only small incremental adjustments were requested by new hearing aid users, suggesting that manufacturers’ default fitting approaches may provide close prescriptive target matching in specific clinical and device contexts [8].

At the same time, hearing aid technology continues to evolve rapidly across successive hearing aid generations. Contemporary flagship hearing aid platforms incorporate increasingly sophisticated technological innovations across both acoustic signal processing and fitting systems, including artificial intelligence (AI)-enabled environmental classification systems, machine learning algorithms, deep neural network-based speech enhancement, and increasingly adaptive amplification strategies [9,10]. Field and laboratory studies of deep-learning-based noise reduction have reported improved real-world listening benefit and, under favorable signal-to-noise conditions, speech intelligibility approaching that of normal-hearing listeners [10,11]. Although these technological developments have primarily been evaluated in relation to speech understanding in noise and listening experiences, they may also afford greater fitting flexibility and facilitate closer prescriptive target matching during best-practice fitting.

Whether these technological advances across successive hearing aid generations translate into closer prescriptive target matching, as distinct from perceptual or self-reported benefit, remains unknown. Previous studies examining hearing aid technology have primarily compared technology tiers (e.g., premium versus basic devices) within a single generation rather than successive hearing aid generations across release years [12,13,14]. Although premium technologies may provide advantages under controlled laboratory conditions, studies have generally demonstrated inconsistent differences in speech understanding, listening effort, sound quality, and real-world outcomes between premium- and basic-level devices [12,13,14]. More recently, a systematic review and meta-analysis found no consistent overall advantage for premium hearing aids across user outcomes, although modest context-specific benefits were observed and only one study reported superior prescriptive target matching for premium devices [15]. Importantly, these studies did not evaluate whether technological progression across successive hearing aid generations influences prescriptive target matching during best-practice fitting.

This question is particularly relevant given the rapid pace of technological progression across successive hearing aid generations. To our knowledge, no previous study has evaluated whether more recently released hearing aid generations achieve closer prescriptive target matching than earlier generations under standardized laboratory conditions. The present secondary analysis addressed this gap by evaluating prescriptive target matching across three technology generations of flagship prescription hearing aids released between 2021 and 2025 under both an unverified Initial Fit and a verified Tuned Fit condition.

## 2. Method

### 2.1. Study Design

This secondary analysis was based on a cross-sectional observational study that evaluated real-ear insertion gain (REIG) relative to NAL-NL2 prescriptive targets across commercially available hearing devices [16]. Standardized laboratory measurements were obtained using a Knowles Electronics Manikin for Acoustic Research (KEMAR) with anthropomorphic pinnae and RA0401 high-frequency ear simulators (GRAS Sound & Vibration, Holte, Denmark). The present analysis was restricted to 29 prescription hearing aids (out of 99 hearing devices that included various prescription and direct-to-consumer hearing devices [16]) to investigate differences in prescriptive target matching across technology generations. The sample size was determined by the number of flagship prescription hearing aids available in the laboratory dataset. No a priori sample size calculation or power analysis was performed.

### 2.2. Data Collection

Detailed descriptions of the data collection procedures have been reported previously [16]. Briefly, standardized REIG measurements were obtained using KEMAR under controlled laboratory conditions following the HearAdvisor testing protocol [17,18]. All prescription hearing aids were evaluated using a standardized N3 moderate sloping sensorineural hearing loss profile [19] and NAL-NL2 prescriptive targets [1].

Devices were assessed under two fitting conditions: an Initial Fit and a Tuned Fit. The Initial Fit represented the manufacturer’s default fitting approach without REM verification. Depending on device capabilities, this included the manufacturer’s recommended first-fit procedure (e.g., audiogram entry, default fitting rationale, acoustic coupling selection, and feedback calibration) or the standard consumer fitting pathway (e.g., app-based setup, in-situ hearing testing, direct audiogram input, or preset/volume adjustment). Initial Fit procedures were standardized across devices using a predefined decision tree implemented by an audiologist. The Tuned Fit represented a best-practice fitting approach in which an audiologist iteratively adjusted available gain parameters and acoustic coupling using the manufacturer’s fitting interface to achieve the closest possible approximation to NAL-NL2 prescriptive targets across 55, 65, and 75 dB SPL input levels while maintaining a stable fit without audible feedback. A successful tuned fit was defined as achieving a Speech Intelligibility Index (SII)-importance-weighted average absolute deviation of <5 dB across 0.5, 1, 2, and 4 kHz, where achievable. Input-level-specific gain adjustments were available for 27 of the 29 devices. For the two devices that did not permit input-level-specific gain adjustment, optimization prioritized the 65 dB SPL condition.

For the present secondary analysis, prescription hearing aids were classified into three technology generations according to the market-release year of the manufacturer’s flagship platform. Flagship devices were defined as the manufacturer’s highest technology-tier prescription hearing aids, incorporating the most recently released processing platform and signal-processing algorithms available at the time of testing. Lower technology tiers were not included in the analysis. Three technology-generation cohorts were defined pragmatically based on year of market introduction: older generation (2021–2022; n = 8), mid generation (2023; n = 11), and newest generation (2024–2025; n = 10). As the dataset included only flagship prescription hearing aids, the analysis reflects technological progression across successive hearing aid generations rather than differences between technology tiers (e.g., basic vs. premium) within a single generation. These year bands were selected to represent successive earlier, intermediate, and more recent flagship platform-release periods while reflecting the distribution of available platforms within the laboratory dataset.

### 2.3. Data Analysis

All statistical analyses were performed in R (version 4.4.2; R Foundation for Statistical Computing, Vienna, Austria) using RStudio (version 2026.01.1+403; Posit Software, PBC, Boston, MA, USA). The primary outcome was root-mean-square error (RMSE) between measured REIG and NAL-NL2 prescriptive targets across six speech frequencies (500, 1000, 2000, 3150, 4000, and 6300 Hz), with lower RMSE values indicating closer agreement with prescriptive targets. RMSE was calculated across six frequencies to provide a broader assessment of prescriptive target matching than the four frequencies included in the tuning criterion. Accordingly, the tuning criterion served to guide fitting optimization, whereas RMSE represented an independent outcome measure of overall target matching across a wider frequency range. Analyses were conducted separately for soft (55 dB SPL), conversational (65 dB SPL), and loud (75 dB SPL) speech input levels under both Initial Fit and Tuned Fit conditions. There were no missing RMSE or release-year data for the 29 included devices. All device-level observations were retained in the analyses; no observations were excluded solely on the basis of statistical outlier screening.

To provide descriptive comparisons across the predefined technology-generation cohorts, Kruskal–Wallis tests were performed for each speech input level and fitting condition as the RMSE values were not normally distributed according to Shapiro-Wilk tests, which indicated significant deviations from normality in 5 of 6 speech input level–by–fitting condition combinations (all *p* < 0.05), with the exception of Initial Fit at 75 dB SPL (*p* = 0.23). Where significant overall differences were observed, pairwise comparisons were conducted using Dunn’s post hoc tests with Holm adjustment for multiple comparisons. Effect size was quantified using epsilon-squared (ε^2^ = [*H* − *k* + 1]/[*N* − *k*]), with values of <0.06, 0.06–0.14, and >0.14 interpreted as small, medium, and large effects, respectively [20,21]. Statistical significance was set at *p* < 0.05.

To account for the clustering of devices within manufacturers, the primary inferential analyses were performed using linear mixed-effects models. Separate models were fitted for each speech input level and fitting condition, with RMSE as the dependent variable, release year as a fixed effect, and manufacturer included as a random intercept. The random-effects structure comprised eleven manufacturer groups, five of which contributed only a single device (Horizon, Jabra/GN, Lucid, Orka, and Rexton). Release year was mean-centred prior to model fitting. Models were fitted using the lme4 [22] and lmerTest [23] packages in R, with degrees of freedom estimated via Satterthwaite’s approximation; the manufacturer random-intercept variance, the intraclass correlation coefficient (ICC), and marginal and conditional R^2^ [24] were calculated using the performance package [25] to quantify the extent of manufacturer-level clustering and overall model fit. Analysing release year as a continuous predictor in the mixed-effects models served as a sensitivity analysis to evaluate whether the findings were robust to the predefined technology-generation categories. Spearman’s rank correlation coefficients with 95% bootstrap confidence intervals were calculated as secondary supportive analyses to examine the association between release year and RMSE.

To assess the potential influence of platform-level pseudoreplication, a sensitivity analysis was conducted by averaging RMSE values across devices sharing the same manufacturer and underlying processing platform within each fitting condition, resulting in one observation per platform. All primary statistical analyses were then repeated using the platform-level dataset. Kruskal–Wallis tests were retained for the platform-level sensitivity analysis for consistency with the primary analyses; Shapiro-Wilk tests on the aggregated platform-level data showed a similar, though less consistent, pattern of non-normality (significant deviations in 2 of 6 speech-level-by-fitting-condition combinations, both *p* < 0.05), which is expected given the reduced sample size (n = 23) and correspondingly lower power to detect non-normality. As an additional check on the robustness of the three-category technology-generation classification, the primary comparisons were repeated using an alternative two-category classification that collapsed devices into an early era (2021–2023, n = 19) and a recent era (2024–2025, n = 10), representing a substantially different boundary placement than the primary classification.

## 3. Results

### 3.1. Sample Characteristics

Twenty-nine flagship prescription hearing aids representing three technology generations (older, n = 8; mid, n = 11; newest, n = 10) were included in the analysis. The 29 prescription hearing aids represented 11 manufacturers. Manufacturer representation varied across technology-generation cohorts, with the older generation including devices from six manufacturers, the mid generation from seven manufacturers, and the newest generation from seven manufacturers (Supplementary Table S1). Several manufacturers contributed multiple devices within the same platform (e.g., Signia IX and Phonak Sphere Infinio), and some corporate groups were represented across multiple brands (e.g., GN: ReSound and Jabra; WS Audiology: Signia and Rexton).

### 3.2. Technology Generation and Prescriptive Target Matching

Following tuning, prescriptive target matching differed across technology generations but not at the manufacturer’s default first-fit settings (Figure 1; Table 1). At the Initial Fit, no significant differences in RMSE were observed between technology generations at any speech input level (all *p* ≥ 0.12). Following tuning, significant differences in RMSE were observed between technology generations at the conversational and loud speech input levels (both *p* < 0.01), with a similar trend observed at the soft speech input level (*p* = 0.055). Following tuning, effect sizes were large at all speech input levels (Table 1).

Post hoc Dunn’s tests with Holm adjustment demonstrated that, under the tuned conversational speech condition (65 dB SPL), the newest technology generation had significantly lower RMSE values than both the older and mid technology generations (both adjusted *p* = 0.015). Under the tuned loud speech condition (75 dB SPL), the newest generation likewise demonstrated significantly lower RMSE values than both the older (adjusted *p* = 0.041) and mid (adjusted *p* = 0.004) technology generations. No significant differences were observed between the older and mid technology generations in either tuned condition (both adjusted *p* > 0.05).

### 3.3. Predictors of Prescriptive Target Matching

Linear mixed-effects models accounting for manufacturer as a random intercept indicated that release year was a significant predictor of prescriptive target matching at conversational speech following tuning (β = −0.15 dB/year, 95% CI [−0.29, −0.01], *p* = 0.048). Directionally similar associations were observed at soft and loud speech input levels, although these did not remain statistically significant after adjustment for manufacturer.

At soft speech, a non-significant trend towards improved prescriptive target matching with more recent release year was observed (β = −0.31 dB/year, 95% CI [−0.64, 0.02], *p* = 0.084). At loud speech, the significant unadjusted association observed in the supportive Spearman analysis (ρ = −0.41, *p* = 0.026) was attenuated after accounting for manufacturer and did not remain significant in the mixed-effects model (β = −0.20 dB/year, 95% CI [−0.46, 0.06], *p* = 0.149). Overall, more recently released hearing aids tended to achieve closer agreement with NAL-NL2 prescriptive targets following tuning across speech input levels, although statistical significance after manufacturer adjustment was observed only at conversational speech. No significant release-year effects were identified for Initial Fit measurements at any speech input level (all *p* ≥ 0.219).

The ICC attributable to manufacturer ranged from 0.04 to 0.55 across the six models (Table 2), indicating that the proportion of RMSE variance attributable to manufacturer-level clustering varied across speech input levels and fitting conditions. Conditional R^2^ exceeded marginal R^2^ across all models, although the additional variance explained by manufacturer differed considerably by condition (Table 2).

Secondary supportive Spearman’s rank correlation analyses demonstrated no significant associations between release year and Initial Fit RMSE across soft, conversational, or loud speech input levels (all *p* ≥ 0.372; *N* = 29). For the Tuned Fit, a non-significant negative association was observed at soft speech (55 dB SPL; ρ = −0.28, 95% CI [−0.60, 0.08], *p* = 0.135; *N* = 29), whereas significant negative associations were observed at conversational speech (65 dB SPL; ρ = −0.50, 95% CI [−0.77, −0.13], *p* = 0.005; *N* = 29) and loud speech (75 dB SPL; ρ = −0.41, 95% CI [−0.69, −0.07], *p* = 0.026; *N* = 29). These findings were generally consistent with the mixed-effects models, demonstrating that more recently released hearing devices tended to achieve closer agreement with NAL-NL2 prescriptive targets following tuning, although statistical significance following adjustment for manufacturer differed across speech input levels. Complete Spearman correlation results are provided in Supplementary Table S2.

### 3.4. Sensitivity Analysis

To evaluate the potential influence of platform-level pseudoreplication arising from multiple form factors sharing the same underlying processing platform, all analyses were repeated after aggregating devices by manufacturer and platform. The sensitivity analyses demonstrated a directionally similar pattern of findings to the primary analyses. Technology-generation differences remained significant for tuned RMSE at soft (*p* = 0.023), conversational (*p* = 0.009), and loud (*p* = 0.005) speech input levels. Similarly, significant associations between release year and tuned RMSE at conversational speech were maintained in both the Spearman correlation (ρ = −0.48, *p* = 0.020) and the linear mixed-effects model (β = −0.12 dB/year, *p* = 0.031), indicating that the association observed for conversational speech in the primary analysis was robust to potential platform-level pseudoreplication. The platform-level analyses additionally demonstrated significant technology-generation differences at soft and loud speech input levels; however, these findings should be interpreted descriptively as they were not adjusted for manufacturer in the same manner as the primary mixed-effects models.

Under the alternative two-category boundary definition (early era, 2021–2023, n = 19, versus recent era, 2024–2025, n = 10), Wilcoxon rank-sum tests showed the same pattern as the primary three-category analysis: no significant differences at the Initial Fit (all *p* ≥ 0.11), and significantly lower RMSE for the recent era at the Tuned Fit across all three speech input levels (soft, *p* = 0.020; conversational, *p* = 0.001; loud, *p* = 0.001), indicating that the direction of the observed technology-generation effects was robust to the alternative technology-generation boundary definition and was not an artifact of the specific three-category classification used in the primary analysis.

## 4. Discussion

The present secondary analysis evaluated whether prescriptive target matching differed across successive technology generations of flagship prescription hearing aids. No technology-generation differences were observed at manufacturers’ unverified Initial Fit settings. Following clinician-driven tuning, newer technology generations demonstrated closer prescriptive target matching across speech input levels, with this association remaining significant after accounting for manufacturer clustering only at conversational speech levels.

The absence of technology-generation differences under Initial Fit conditions indicates that technological progression across successive technology generations does not automatically translate into improved target matching at default settings. These findings are consistent with prior evidence demonstrating that standard manufacturer default algorithms typically under-amplify high frequencies relative to validated prescriptive targets [8]. Although contemporary fitting software can approximate targets reasonably well in specific contexts, default manufacturer fitting algorithms are designed to balance audibility and listening comfort and may therefore deviate from validated prescriptive targets [8]. The present findings suggest that advances in hearing aid technology do not inherently improve prescriptive target matching under Initial Fit conditions. Instead, they highlight that REM verification and clinician-driven tuning remain integral components of best-practice hearing aid fitting, including for the most recently released devices.

The closer prescriptive target matching observed in newer technology generations following clinician-driven tuning suggests that technological progression may facilitate closer approximation to prescribed gain targets when clinician-driven tuning is performed. Although the present study cannot determine which specific technological developments contributed to the observed association, several plausible mechanisms may explain why newer hearing aid generations achieved closer prescriptive target matching following clinician-driven tuning. Improvements in feedback management may permit higher levels of high-frequency amplification before acoustic feedback occurs, thereby providing greater flexibility to achieve prescriptive targets [26]. In addition, more advanced fitting software and increased configurability of fitting interfaces may enable clinicians to make finer frequency-specific and input-level adjustments, facilitating closer approximation to prescribed gain [27]. Whether the observed association primarily reflects advances in hearing aid signal-processing capabilities, fitting software, or a combination of both cannot be determined from the present study and warrants further investigation.

Interestingly, the association between technology generation and prescriptive target matching remained statistically significant only at conversational speech levels after accounting for manufacturer clustering. This pattern coincided with marked differences in manufacturer-level clustering across speech input levels. Manufacturer-level clustering was minimal at conversational speech but substantially greater at soft and loud speech levels, indicating that between-manufacturer differences accounted for a larger proportion of RMSE variance under these fitting conditions. Consequently, manufacturer-level variability may have had a greater influence on the mixed-effects estimates at soft and loud speech levels than at conversational speech. Further studies including larger numbers of devices across manufacturers are needed to clarify the relative contributions of technology generation and manufacturer to prescriptive target matching across speech input levels.

The persistence of the association at conversational speech levels may be particularly clinically relevant, given that conversational-level speech represents a substantial proportion of everyday listening environments. Previous research has demonstrated that REM-verified fittings are associated with statistically significant improvements in speech intelligibility in quiet and noise, self-reported listening abilities, and user preference relative to manufacturers’ default fitting approaches [2]. However, the magnitude and clinical significance of these benefits remain uncertain [2]. Whether the relatively small differences in prescriptive target matching observed between technology generations in the present study, equating to an estimated reduction in RMSE of approximately 0.60 dB between the oldest and newest hearing aid generations, translate into meaningful improvements in speech understanding, listening experiences, or patient-reported outcomes therefore also remains unclear. The observed reduction in RMSE should not be interpreted as evidence of superior patient outcomes or as justification for selecting or upgrading a hearing aid solely on the basis of technology generation. Further research is needed to determine whether the modest improvements in target matching afforded by newer hearing aid generations are associated with measurable benefits in real-world hearing aid outcomes.

Several limitations should be considered when interpreting these findings. The sample comprised 29 flagship prescription hearing aids from 11 manufacturers, and no a priori sample size calculation was performed because the sample was determined by the eligible devices available in the existing laboratory dataset. Consequently, the study may have had limited power to detect smaller technology-generation effects, particularly at soft and loud speech levels, and the estimates should be interpreted with appropriate caution. Measurements were obtained under standardized laboratory conditions using a KEMAR manikin and a single moderate sloping sensorineural hearing loss profile; although this controlled device-level comparisons, it did not capture variability in individual ear acoustics, audiometric configurations, clinical fittings, or real-world listening conditions. Additionally, two devices did not permit input-level-specific gain adjustments, with tuning therefore prioritizing the 65 dB SPL condition, which may have influenced comparisons across input levels. As RMSE included two frequencies beyond the fitting criterion, deviations at 3150 and 6300 Hz may have influenced overall RMSE values. Furthermore, the analysis was restricted to flagship prescription hearing aids, limiting generalizability to commonly prescribed mid-level and entry-level devices. Release year was also used as a proxy for technological progression, precluding conclusions regarding specific technological innovations or other fitting pathways. Finally, although the conversational-speech finding was robust to the sensitivity analyses performed, the present study evaluated prescriptive target matching rather than patient outcomes. Whether the modest improvements observed in more recently released hearing aid generations translate into meaningful benefits in speech understanding, listening experiences, or patient-reported outcomes remains to be established. Only NAL-NL2 targets were evaluated; the findings may not generalize to other prescriptive procedures, including DSL. Future studies should examine DSL target matching, particularly for pediatric fittings, using age-appropriate audiometric profiles and clinical verification procedures. Finally, the authors’ disclosed commercial relationships should be considered when interpreting the findings.

## 5. Conclusions

Newer technology generations of flagship prescription hearing aids demonstrated closer prescriptive target matching following clinician-driven tuning, although no technology-generation differences were observed at manufacturers’ unverified Initial Fit settings. After accounting for manufacturer clustering, this effect remained significant only at conversational speech levels. These findings suggest that technological progression may increase the potential for achieving closer prescriptive target matching during best-practice hearing aid fitting procedures rather than inherently improving target matching under default fitting conditions. REM verification and clinician-driven tuning remain fundamental to achieving prescribed amplification targets irrespective of technology generation. Whether these modest improvements in target matching translate into clinically meaningful benefits for hearing aid users remains to be established.

## Figures and Tables

**Figure 1:**
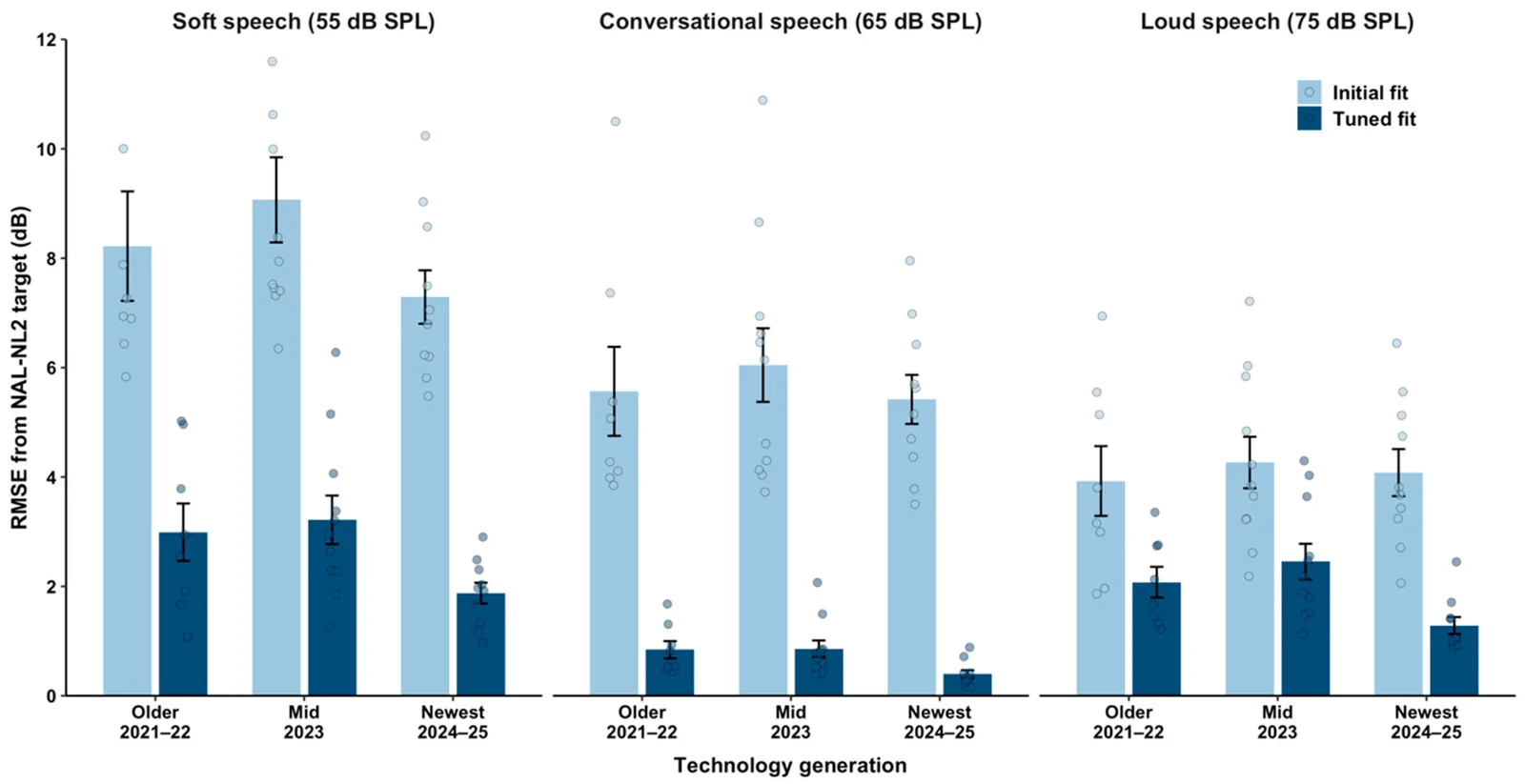
Mean RMSE (dB) between measured REIG and NAL-NL2 prescriptive targets across technology generations (older, n = 8; mid, n = 11; newest, n = 10) for Initial Fit and Tuned Fit conditions. Error bars represent ± SEM, and points represent individual devices. RMSE = root-mean-square error; REIG = real-ear insertion gain; NAL-NL2 = National Acoustic Laboratories–Non-Linear 2; SEM = standard error of the mean.

**Table 1:** Mean (SD) RMSE (dB) from NAL-NL2 Prescriptive Targets by Technology Generation, Fitting Condition, and Speech Input Level.

	Older (2021–2022) n = 8	Mid (2023) n = 11	Newest (2024–2025) n = 10	Kruskal–Wallis H(2)	*p*	ε^2^
Initial Fit						
55 dB SPL	8.22 (2.83)	9.07 (2.58)	7.29 (1.54)	4.26	0.119	0.087
65 dB SPL	5.57 (2.30)	6.05 (2.23)	5.42 (1.42)	0.50	0.779	0.000
75 dB SPL	3.93 (1.80)	4.26 (1.56)	4.08 (1.36)	0.52	0.770	0.000
Tuned Fit						
55 dB SPL	2.99 (1.49)	3.22 (1.47)	1.88 (0.61)	5.80	0.055	0.146
65 dB SPL	0.84 (0.45)	0.86 (0.50)	0.39 (0.23)	10.33	0.006 *	0.321
75 dB SPL	2.08 (0.79)	2.45 (1.08)	1.28 (0.49)	11.03	0.004 *	0.347

Note. RMSE = root-mean-square error; NAL-NL2 = National Acoustic Laboratories–Non-Linear 2. * *p* < 0.05. Effect size (ε^2^) and statistical significance (*p*-value) quantify different aspects of the data and need not necessarily agree. ε^2^ values below 0 (when *H* < *k* − 1) are reported as 0.

**Table 2:** Linear Mixed-Effects Model Results for Release Year Predicting RMSE, by Fitting Condition and Speech Input Level.

Fitting Condition	Speech Level	β (dB/Year), 95% CI	*p*	Manufacturer Variance (τ00)	ICC	Marginal R^2^	Conditional R^2^
Initial Fit	55 dB SPL	−0.46 [−1.16, 0.25]	0.219	1.34	0.23	0.04	0.27
	65 dB SPL	−0.23 [−0.82, 0.37]	0.463	0.86	0.22	0.02	0.23
	75 dB SPL	−0.01 [−0.48, 0.47]	0.974	0.42	0.17	0.00	0.17
Tuned Fit	55 dB SPL	−0.31 [−0.64, 0.02]	0.084	1.12	0.55	0.06	0.58
	65 dB SPL	−0.15 [−0.29, −0.01]	0.048 *	0.01	0.04	0.13	0.17
	75 dB SPL	−0.20 [−0.46, 0.06]	0.149	0.45	0.45	0.05	0.48

Note. RMSE = root-mean-square error; ICC = intraclass correlation coefficient attributable to manufacturer; τ00 = manufacturer random-intercept variance. Release year was mean-centred (*M* = 2023.07) prior to model fitting. Degrees of freedom were estimated using Satterthwaite’s approximation (lmerTest). * *p* < 0.05.

## Data Availability

The datasets generated and/or analyzed for the current study are available from the corresponding author upon reasonable request.

## Supplementary Materials

The following supporting information can be downloaded at: https://www.mdpi.com/article/10.3390/audiolres16050145/s1, Table S1. Prescription hearing aids included in the secondary analysis, classified by manufacturer, platform, release year, and technology generation; Table S2. Spearman Correlations.
