# Supplementary material for: Prescriptive Target Matching Across Hearing Aid Technology Generations: A Real-Ear Insertion Gain Analysis
Source: Audiol Res. 2026 Sep 25;16(5):145. doi: 10.3390/audiolres16050145 (PMC13701747; doi:10.3390/audiolres16050145)
Supplement: Supplementary file 1 [file audiolres-16-00145-s001.zip › audiolres-4544766-supplementary.pdf]

Supplementary Table S1. Prescription hearing aids included in the secondary analysis, classified by manufacturer, platform, release year, and technology generation.

Product	Manufacturer	Platform	Release Year	Technology Generation
**Older Generation (n = 8)**				
Oticon More 1 miniRITE 312	Oticon	More	2021	Older
Signia Styletto 7 AX	Signia	AX	2021	Older
Starkey Evolv AI 2400 RIC RT	Starkey	Evolv AI	2021	Older
Oticon Real 1 miniRITE 312	Oticon	Real	2022	Older
Phonak Audeo Lumity RT 90	Phonak	Lumity	2022	Older
ReSound Omnia 9 RIE961	ReSound	Omnia	2022	Older
ReSound Omnia 9 RU960	ReSound	Omnia	2022	Older
Widex Moment SmartRIC 440	Widex	Moment SmartRIC	2022	Older
**Mid Generation (n = 11)**				
Jabra Enhance Pro 20 Micro RIE	Jabra/GN	Enhance Pro 20	2023	Mid
Lucid Ultra 128*	Lucid	Ultra	2023	Mid
Orka Two	Orka	Two	2023	Mid
ReSound Nexia 9 60S microRIE	ReSound	Nexia	2023	Mid
Signia ActivePro IX	Signia	IX	2023	Mid
Signia Pure Charge&Go BCT 7 IX	Signia	IX	2023	Mid
Signia Pure Charge&Go T IX 7	Signia	IX	2023	Mid
Signia Silk Charge&Go IX	Signia	IX	2023	Mid
Signia Styletto Charge&Go IX 7	Signia	IX	2023	Mid
Starkey Genesis AI 24 RIC	Starkey	Genesis AI	2023	Mid
Widex Moment Sheer sRIC R D 440	Widex	Moment Sheer	2023	Mid
**Newest Generation (n = 10)**				
Hear Horizon Go IX	Horizon	Go IX	2024	Newest
Oticon Intent 1 miniRITE	Oticon	Intent	2024	Newest
Phonak Audeo R Infinio 90	Phonak	Infinio	2024	Newest
Phonak Audeo Sphere Infinio 90	Phonak	Sphere Infinio	2024	Newest
Phonak Sphere Infinio Ultra	Phonak	Sphere Infinio	2024	Newest
Rexton Reach R-Li T80*	Rexton	Reach	2024	Newest
Starkey Omega AI 24 RIC	Starkey	Omega AI	2024	Newest
ReSound Vivia 9 60S microRIE	ReSound	Vivia	2025	Newest
Starkey Edge AI RIC	Starkey	Edge AI	2025	Newest
Widex Allure RIC R D 440	Widex	Allure	2025	Newest

*Input-level-specific gain adjustments were not available for these devices.

Supplementary Table S2. Spearman Correlations

Fit condition	Input level	n	Spearman's rho	95% CI	p-value
**Initial**	55 dB SPL	29	-0.17	[-0.57, 0.28]	.372
**Initial**	65 dB SPL	29	0.03	[-0.37, 0.43]	.889
**Initial**	75 dB SPL	29	0.11	[-0.30, 0.50]	.568
**Tuned**	55 dB SPL	29	-0.28	[-0.60, 0.08]	.135
**Tuned**	65 dB SPL	29	-0.50	[-0.77, -0.13]	.005
**Tuned**	75 dB SPL	29	-0.41	[-0.69, -0.07]	.026
